# Human *Salmonella* Typhi exposure generates differential multifunctional cross‐reactive T‐cell memory responses against *Salmonella* Paratyphi and invasive nontyphoidal *Salmonella*

**DOI:** 10.1002/cti2.1178

**Published:** 2020-09-24

**Authors:** Rekha R Rapaka, Rezwanul Wahid, Stephanie Fresnay, Jayaum S Booth, Thomas C Darton, Claire Jones, Claire S Waddington, Myron M Levine, Andrew J Pollard, Marcelo B Sztein

**Affiliations:** ^1^ Center for Vaccine Development and Global Health University of Maryland School of Medicine Baltimore MD USA; ^2^ Department of Medicine University of Maryland School of Medicine Baltimore MD USA; ^3^ Department of Pediatrics University of Maryland School of Medicine Baltimore MD USA; ^4^ Oxford Vaccine Group Department of Paediatrics University of Oxford and the NIHR Oxford Biomedical Research Centre Oxford UK; ^5^ Present address: Stephanie Fresnay GlaxoSmithKline Rockville MD USA; ^6^ Present address: Thomas C Darton University of Sheffield Medical School Sheffield UK; ^7^ Present address: University of Cambridge Cambridge UK

**Keywords:** human challenge, iNTS, invasive *Salmonella*, Paratyphi, *S.* Typhi, T‐cell memory

## Abstract

**Objective:**

There are no vaccines for most of the major invasive *Salmonella* strains causing severe infection in humans. We evaluated the specificity of adaptive T memory cell responses generated after *Salmonella* Typhi exposure in humans against other major invasive *Salmonella* strains sharing capacity for dissemination.

**Methods:**

T memory cells from eleven volunteers who underwent controlled oral challenge with *wt* *S*. Typhi were characterised by flow cytometry for cross‐reactive cellular cytokine/chemokine effector responses or evidence of degranulation upon stimulation with autologous B‐lymphoblastoid cells infected with either *S*. Typhi, *Salmonella* Paratyphi A (PA), *S*. Paratyphi B (PB) or an invasive nontyphoidal *Salmonella* strain of the *S*. Typhimurium serovar (iNTSTy).

**Results:**

Blood T‐cell effector memory (T_EM_) responses after exposure to *S*. Typhi in humans evolve late, peaking weeks after infection in most volunteers. Induced multifunctional CD4^+^ Th1 and CD8^+^ T_EM_ cells elicited after *S*. Typhi challenge were cross‐reactive with PA, PB and iNTSTy. The magnitude of multifunctional CD4^+^ T_EM_ cell responses to *S*. Typhi correlated with induction of cross‐reactive multifunctional CD8^+^ T_EM_ cells against PA, PB and iNTSTy. Highly multifunctional subsets and T central memory and T effector memory cells that re‐express CD45 (T_EMRA_) demonstrated less heterologous T‐cell cross‐reactivity, and multifunctional Th17 elicited after *S*. Typhi challenge was not cross‐reactive against other invasive *Salmonella*.

**Conclusion:**

Gaps in cross‐reactive immune effector functions in human T‐cell memory compartments were highly dependent on invasive *Salmonella* strain, underscoring the importance of strain‐dependent vaccination in the design of T‐cell‐based vaccines for invasive *Salmonella*.

## Graphical abstract

Vaccines are lacking against several of the major causes of invasive *Salmonella* disease, such as invasive nontyphoidal *Salmonella* and *S. *Paratyphi A. These studies demonstrate that T memory cell effector responses generated after human *wt* *Salmonella* Typhi mucosal infection are multifunctional and cross‐reactive against *S*. Paratyphi A, *S*. Paratyphi B and an invasive nontyphoidal *Salmonella* Typhimurium strain, yet the specific functional characteristics of the memory T‐cell responses are significantly impacted by serovar. These data provide a rationale for strain‐specific vaccination against invasive *Salmonella* for next‐generation T‐cell‐based vaccines.

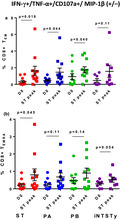

## Introduction

Invasive *Salmonella* infection, caused by typhoidal *Salmonella* (most commonly *S*. Typhi or *S*. Paratyphi A) or invasive nontyphoidal *Salmonella* (iNTS), is spread by faecal–oral route and is responsible for millions of infections yearly and over 600 000 deaths annually, with most mortality caused by iNTS.^1^, ^2^, ^3^ There are no vaccines in clinical use known to prevent disease from *S*. Paratyphi A (PA) or iNTS. Dominant *Salmonella* Typhimurium and *S*. Enteriditis iNTS strains evolving within the last 60 years, identified in sub‐Saharan Africa, have significant genomic changes associated with invasive disease absent from typical nontyphoidal *Salmonella* (NTS) strains producing localised gastroenteritis.^4^, ^5^, ^6^ iNTS disease, often presenting with bacteraemia and focal infection such as meningitis or pneumonia, is associated with a case fatality rate of over 20% and is seen in young children with risk factors such as malnutrition and anaemia, or in the setting of co‐infections with malaria or HIV.^7^, ^8^ Three serovars, *S*. Typhi, PA and *S*. Paratyphi B (PB), are the predominant causes of enteric fever (typhoid and paratyphoid disease) globally, and genomic differences between typhoidal serovars are underscored by studies demonstrating their convergent evolution.^9^, ^10^, ^11^ PA incidence is rapidly increasing in South‐East Asia, and an epidemic of extensively drug‐resistant *S*. Typhi is ongoing in South Asia since 2016.^12^, ^13^, ^14^ Given high incidence of invasive *Salmonella* infection, low‐sensitivity diagnostics and high rates of antimicrobial use for suspected infection, antimicrobial resistance against invasive *Salmonella* is increasing.^12^, ^13^, ^15^, ^16^, ^17^, ^18^ Improving vaccination against all major invasive *Salmonella* by preventing severe and fatal infections would counteract the threat posed by emerging antibiotic‐resistant strains of *Salmonella* and decrease mortality associated with these infections.

While there are data that invasive *Salmonella* host defence in humans involves antibody production,^19^ the importance of T‐cell‐dependent host defence mechanisms against these intracellular bacteria is supported by studies demonstrating increased human susceptibility to invasive *Salmonella* disease in the presence of specific STAT4 or HLA II polymorphisms or disruptions in IL‐12 or IL‐23 signalling pathways in humans.^20^, ^21^, ^22^ Murine models of invasive *Salmonella* infection additionally demonstrate a critical role for T cells in host defence.^23^ Studies in a human challenge model in which volunteers were orally challenged with wild‐type *S*. Typhi demonstrate that high baseline frequencies of multifunctional CD8^+^ T_EM_ cell responses correlated with protection from the development of typhoid disease.^24^ *Salmonella* Typhi vaccines administered in humans generate CD8^+^ T cells with the capacity to kill *Salmonella*‐infected cells *in vitro*, with killing function correlating with expression of CD8^+^ T‐cell IFN‐γ production.^25^, ^26^, ^27^ HIV infection and decreased CD4^+^ T‐cell counts correlate with susceptibility to invasive nontyphoidal *Salmonella* infection rather than disease with typhoidal strains.^28^ These observations demonstrate that the characteristics of human T‐cell responses and the genetics of the *Salmonella* strain fundamentally impact risk of invasive *Salmonella* infection in humans.

There are three vaccines currently in clinical use against *S*. Typhi, but two of these are based on the Vi capsular polysaccharide found in *S*. Typhi and absent in other major prevalent invasive *Salmonella* strains. The live‐attenuated Ty21a vaccine (generated from an *S*. Typhi strain by nondirected mutagenesis) does not confer cross‐protection against PA disease,^29^ but was moderately protective against PB.^30^ After human Ty21a vaccination, low frequencies of highly multifunctional PA cross‐reactive peripheral CD8^+^ T cells are elicited compared to frequencies of reactive CD8^+^ T cells against *S*. Typhi.^31^ It is unknown whether wild‐type *S*. Typhi contains antigens able to elicit both cross‐reactive CD4^+^ and CD8^+^ T‐cell effector functions against other major invasive *Salmonella* for which vaccines are lacking, such as iNTS strains and PA, or how biologic differences between different invasive *Salmonella* strains impact T‐cell priming and the development of T‐cell memory and effector functions in humans.

Mice inherently resist mucosal and systemic infection with *S*. Typhi and develop systemic infection with *Salmonella* strains that typically cause only local gastroenteritis in humans. Given that host species and *Salmonella* strain are important in host susceptibility to disease and disease pathogenesis, we sought to evaluate elements of T‐cell cross‐reactivity in humans against invasive *Salmonella*. The human challenge model directs acquisition of infection through oral–mucosal exposure and characterisation of systemic memory immune responses in individuals longitudinally, without previous history of invasive *Salmonella* infection. Here, in a controlled human oral challenge model of *S*. Typhi infection, we dissect the cross‐reactive immune effector functions of induced memory T cells within different human memory T‐cell subsets, against PA, PB and an *S*. Typhimurium isolated in Mali of the ST313 multilocus sequence type (iNTSTy), as a representative iNTS of serovar Typhimurium circulating in sub‐Saharan Africa.

## Results

Volunteers (*n* = 11) developed typhoid disease approximately 9 days after oral *wt* *S*. Typhi challenge. On average, volunteers exhibited classic clinical features of acute systemic *S*. Typhi infection such as decreases in platelets and serum haemoglobin, as summarised in Supplementary table 1. At the time of typhoid diagnosis, volunteers immediately started a 2‐week curative antibiotic regimen for *S*. Typhi infection.

**Figure 1:**
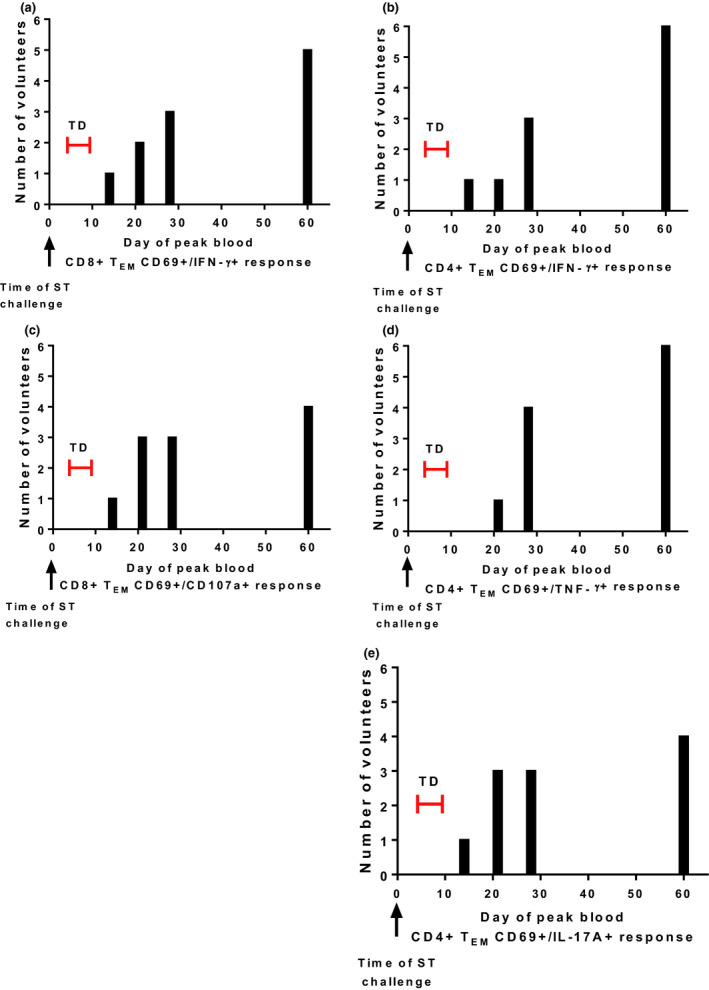
Late development of peak peripheral blood T‐cell effector memory responses in humans orally challenged with *wt* *Salmonella* Typhi. CD8^+^ T_EM_ or CD4^+^ T_EM_ cells from individual volunteers were evaluated for CD107a expression, and production of IFN‐γ, TNF‐α or IL‐17A upon stimulation with autologous *S*. Typhi (ST)‐infected target cells on days 0, 14, 21, 28 and 60 days after challenge. Background responses from uninfected targets were subtracted. The timepoint of highest effector responses per volunteer (*n* = 11) per effector function was determined for **(a)** CD8^+^/CD69^+^/IFN‐γ^+^ T_EM _cells, **(b)** CD8^+^/CD69^+^/CD107a^+^ T_EM _cells, **(c)** CD4^+^/CD69^+^/IFN‐γ^+^ T_EM_ cells, **(d)** CD4^+^/CD69^+^/TNF‐α^+^ T_EM_ cells or **(e)** CD4^+^/CD69^+^/IL‐17A^+^ T_EM _cells. Each measurement was performed once per volunteer per effector function. TD: typhoid disease, red bar indicating range of days observed in volunteers for time of TD diagnosis. Volunteers initiated a curative antibiotic course at the time of TD diagnosis.

Peripheral blood mononuclear cells (PBMCs) collected from volunteers prior to challenge (day 0), and at days 14, 21, 28 and 60 after challenge, were stimulated with autologous B‐LCL infected with *S*. Typhi. Frequencies of activated CD8^+^ T effector memory cells (CD8^+^ T_EM_, CD 69^+^/CD62L^−^/CD45RA^−^) were analysed for expression of CD107a or IFN‐γ production. Net responses were subtracted from responses to uninfected B‐LCL at all timepoints to determine the peak response per cytokine or effector function. Eight of 11 volunteers had peak IFN‐γ effector responses 28 days or later after challenge, and 7 of 11 volunteers had peak frequencies of *S*. Typhi‐reactive CD8^+^ T_EM_ cell CD107a expression (a degranulation marker) 28 days or later after challenge (Figure 1a and c). Activated CD4^+^ T_EM_ cell responses (CD4^+^ T_EM_, CD69^+^/CD62L^‐^/CD45RA^‐^) were also evaluated following stimulation with *S*. Typhi‐infected autologous B‐LCL for individual frequencies of IFN‐γ, TNF‐α or IL‐17A expression (Figure 1b, d and e). For these cell subsets as well, most volunteers develop a peak response for these individual effector functions 28 days or later after challenge, occurring in 9 of 11 volunteers for activated CD4^+^ T_EM_ cells expressing IFN‐γ. Peak blood CD4^+^ and CD8^+^ T‐cell responses after *S*. Typhi challenge occurred long after the diagnosis of typhoid disease and after completion of antibiotic treatment in most volunteers (Figure 1).

**Figure 2:**
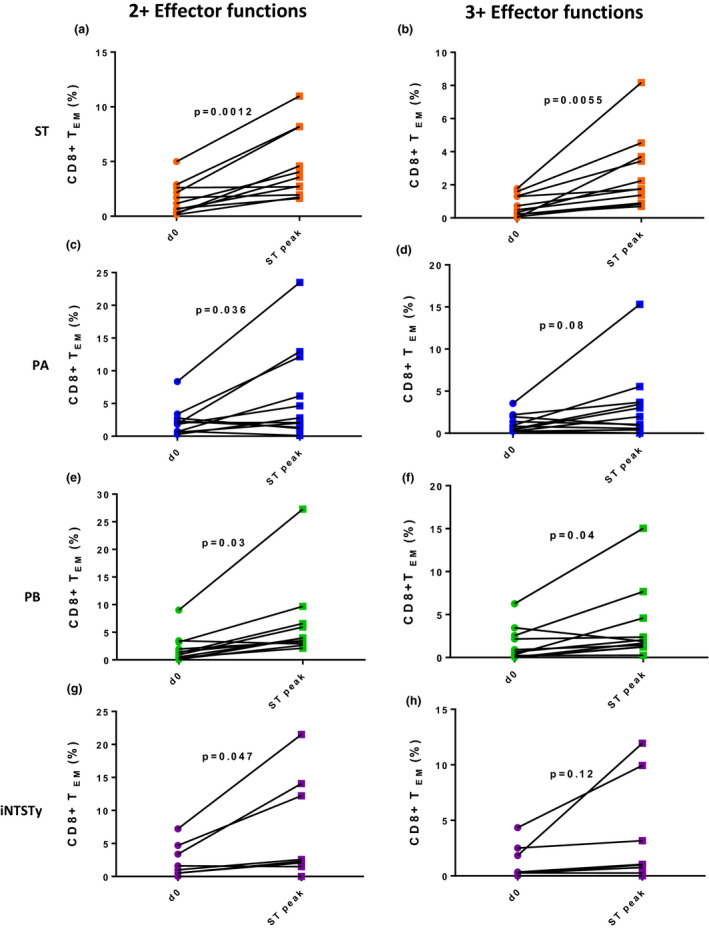
Oral *wt* *Salmonella* Typhi challenge in humans induces multifunctional CD8^+^ T_EM_ cells that are cross‐reactive with other *Salmonella* serovars. PBMCs from individual volunteers collected prior to (d0) and at the peak of the total CD8^+^ T_EM_ IFN‐γ response after *S*. Typhi challenge (ST peak) were stimulated with targets infected with either ST **(a, b)**, PA **(c, d)**, PB **(e, f)** or iNTSTy **(g, h)**. CD8^+^ T_EM_ cells were analysed for simultaneous expression of the following effector functions (production of TNF‐α, IL‐17A, IFN‐γ, MIP‐1β, IL‐2 and/or expression of CD107a). Frequencies of reactive multifunctional CD69^+^ CD8^+^ T_EM_ cells with at least two concomitant effector functions **(a, c, e, g)**; and multifunctional CD69^+^ CD8^+^ T_EM_ cells exhibiting at least three concomitant effector functions **(b, d, f, h)**. *n* = 11 volunteers for **a**–**f**, *n* = 8 volunteers for **g** and **h**. Paired responses from each volunteer are indicated by lines, and one measurement was performed for each volunteer per timepoint. Data were analysed by a paired Student's *t*‐test; *P*‐values are displayed.

We next assessed for the presence of cross‐reactive T‐cell responses against other invasive *Salmonella* elicited after *S*. Typhi challenge. PBMCs from individual volunteers collected prior to or at various timepoints after challenge were stimulated with autologous B‐LCL‐infected with either *S*. Typhi (ST), *S*. Paratyphi A (PA), *S*. Paratyphi B (PB) or *S*. Typhimurium iNTS with the ST313 genotype (sub‐Saharan African iNTS, iNTSTy). A representative volunteer's CD4^+^ T_EM_ and CD8^+^ T_EM_ cell effector responses (expression of TNF‐α, IFN‐γ, IL‐2, MIP‐1β, IL‐17A or surface CD107a expression) are shown over time against four different invasive *Salmonella* strains, as measured following stimulation of B‐LCL‐infected targets (Supplementary figure 1). For each of the 11 volunteers, the timepoint of the peak of the CD8^+^ T_EM_ cells IFN‐γ response was identified against ST (ST peak). At ST peak, CD8^+^ T_EM_ cells were evaluated for simultaneous expression of two or more effector functions upon stimulation with B‐LCL infected with either PA, PB or iNTSTy. Volunteers demonstrated an induced CD8^+^ T_EM_ cell cross‐reactive response, with high frequencies of multifunctional CD8^+^ T_EM_ cells with two or more concomitant effector functions against typhoidal and invasive nontyphoidal *Salmonella* (Figure 2a, c, e and g). When these same CD8^+^ T_EM_ cells were evaluated for reactivity against PA, PB or iNTSTy with 3 or more concomitant effector functions, we observed that while there was an induced cross‐reactive response against PB, there were limited induced cross‐reactive responses against PA or iNTSTy (Figure 2b, d, f and h). These data suggest that while multifunctional cross‐reactive CD8^+^ T_EM_ cells are induced against other invasive *Salmonella* after oral *S*. Typhi challenge in humans, highly multifunctional CD8^+^ T_EM_ subset responses occur only against *S*. Typhi and *S*. Paratyphi B.

**Figure 3:**
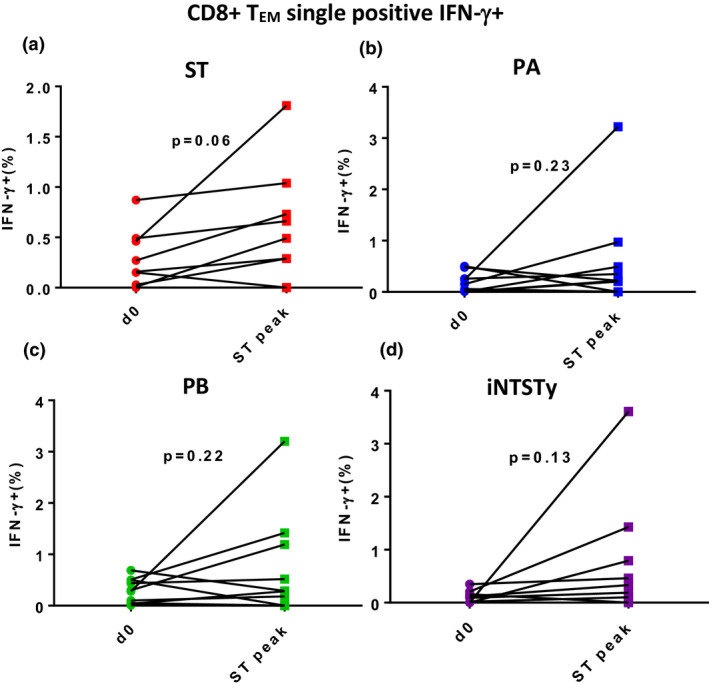
Lack of induction of serovar cross‐reactive CD8^+^ T_EM_ cells producing IFN‐γ in the absence of simultaneous production of either TNF‐α, MIP‐1β, IL‐17A, IL‐2 or surface expression of CD107a in the blood of *Salmonella* Typhi‐challenged volunteers. PBMCs from *S*. Typhi‐challenged participants were stimulated with target cells infected with either **(a)** ST, **(b)** PA, **(c)** PB or **(d)** iNTSTy. Boolean gating was performed on CD69^+^CD8^+^ T_EM_ cells to evaluate individual cell expression of IFN‐γ in the absence of other cytokines above or expression of CD107a in individual cells. Frequencies are shown for CD8^+^ T_EM_ cells prior to challenge (d0) and at the peak of the CD8^+^ T_EM_ cell IFN‐γ response (ST peak). *n* = 11 volunteers for **a–c**, *n* = 8 volunteers for **d**. Paired responses are indicated by lines, and one measurement was performed for each volunteer per timepoint. Data were analysed by a paired Student's *t*‐test; *P*‐values are displayed.

Given the strong correlation of human CD8^+^ T‐cell production of IFN‐γ against *Salmonella*‐infected cells and human *in vitro* cytolytic activity of *Salmonella*‐infected cells,^26^ we assessed the hypothesis that CD8^+^T_EM_ cells induced after *S*. Typhi challenge predominate in IFN‐γ^+^ multifunctional subsets. To this end, we measured frequencies of CD8^+^ T_EM_ cells expressing IFN‐γ alone, that is, not simultaneously expressing any of the other measured effector functions (TNF‐α, IL‐2, MIP‐1β, IL‐17A or surface CD107a). At the peak of the CD8^+^ T_EM_ cell response against *S*. Typhi, although a trend was apparent, we found that CD8^+^ T_EM_ cells expressing only IFN‐γ were not significantly induced after oral challenge (Figure 3a). In addition, there was no significantly induced cross‐reactive response of cells with this phenotype against other typhoidal or invasive nontyphoidal *Salmonella* strains (Figure 3b–d).

**Figure 4:**
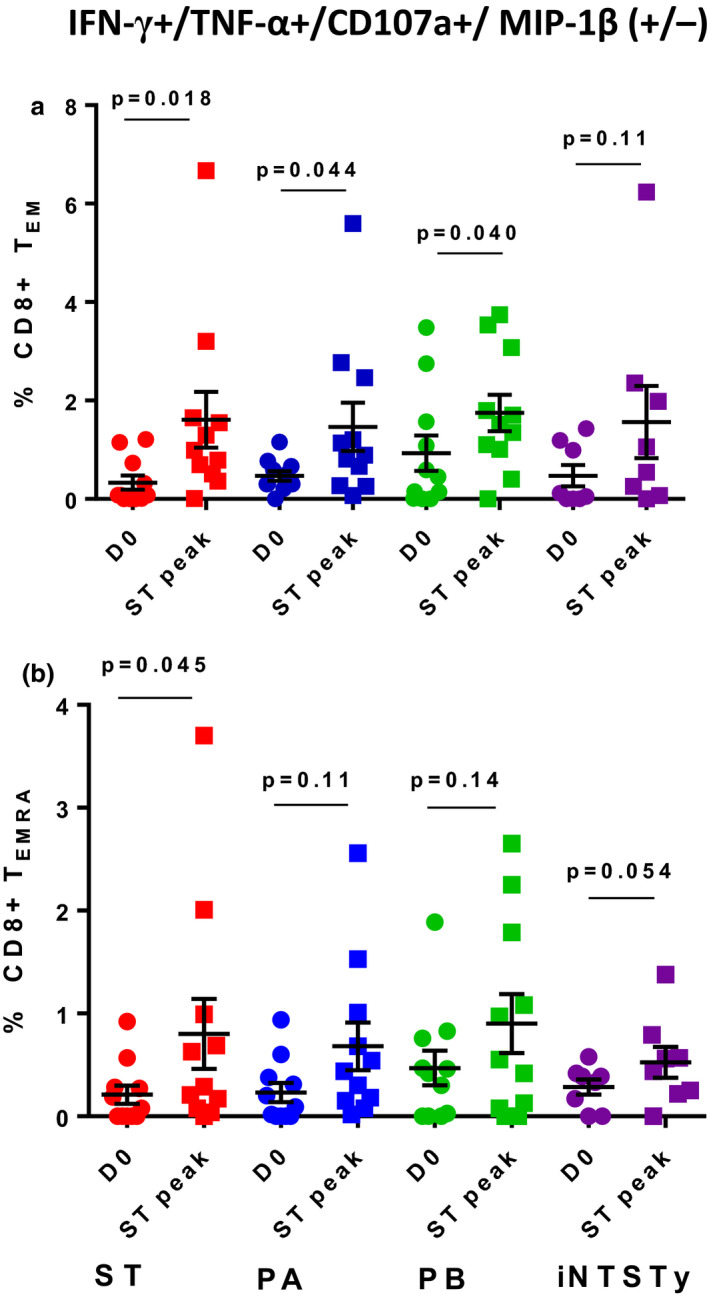
Induction of multifunctional, serovar cross‐reactive blood CD8^+^ T_EM_ cells, but not CD8^+^ T_EMRA_ cells that simultaneously produce IFN‐γ and TNF‐α and express CD107a, with or without MIP‐1β production, after *Salmonella* Typhi challenge. PBMCs prior to infection (d0) and at the timepoint of peak total peripheral CD69^+^ CD8^+^ T_EM_ cell IFN‐γ response after *S*. Typhi challenge in each volunteer (ST peak), were stimulated with autologous targets infected with either ST, PA, PB or iNTSTy. CD69^+^ CD8^+^ T_EM_ cells **(a)** or CD69^+^ CD8^+^ T_EMRA_ cells **(b)** were analysed for simultaneous expression of 6 effector functions (production of TNF‐α, IL‐17, IFN‐γ, MIP‐1β, IL‐2 and/or expression of CD107a). Mean ± SEM and individual responses are shown. *n* = 11 volunteers for ST, PA and PB reactivity, *n* = 8 volunteers for iNTSTy reactivity. One measurement was performed per volunteer. Data were analysed by a paired Student's *t*‐test; *P*‐values are displayed.

In contrast, high frequencies of CD8^+^ T_EM_ cells simultaneously producing IFN‐γ and TNF‐α, and expressing the degranulation marker CD107a, with or without simultaneous expression of MIP‐1β, were induced following *S*. Typhi challenge. These induced responses were significantly cross‐reactive against PA and PB, but not against the more genetically distant iNTSTy (Figure 4a). Of note, this multifunctional CD8^+^ T_EM_ population induced by human *S*. Typhi challenge is of higher magnitude than the induced CD8^+^ T_EMRA_ population, identified as CD62L^−^/CD45RA^+^ (Figure 4b). The CD8^+^ T_EMRA_ population is notable for re‐expression of CD45RA and is a more highly differentiated human T memory cell population, with lower proliferative capacity, than CD8^+^ T_EM_ cells. We have previously shown that multifunctional *S*. Typhi‐specific CD8^+^ T_EMRA_ cells are induced after Ty21a vaccination^31^ and also after human oral *S*. Typhi challenge,^24^ albeit at lower frequencies than those observed in the CD8^+^ T_EM_ population. Here, we observed significant increases in the frequencies of this population against ST, but no significant induction of cross‐reactive CD8^+^ T_EMRA_ cells with the IFN‐γ^+^/TNF‐α^+^/CD107a^+^/MIP‐1β^+/−^ phenotype against PA, PB or iNTS. (Figure 4b). Hence, this multifunctional CD8^+^ T_EMRA_ subset elicited after human oral *S*. Typhi challenge is induced at lower frequencies and is minimally cross‐reactive with other invasive *Salmonella*.

**Figure 5:**
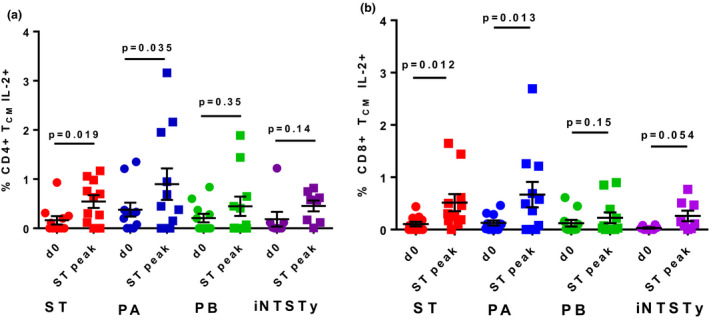
Increased frequencies of T_CM_ cells expressing IL‐2 that are cross‐reactive with *Salmonella* Paratyphi A after *Salmonella* Typhi challenge. PBMCs prior to infection (d0) or at the timepoint of peak total peripheral CD8^+^ T_EM_ or CD4^+^ T_EM_ IFN‐γ responses after *S*. Typhi challenge per individual volunteer (ST peak) were stimulated with targets infected with either ST, PA, PB, or iNTSTy. CD69^+^ CD4^+^ T_CM_ **(a)** and CD69^+^ CD8^+^ T_CM_ **(b)** cells were assessed for intracellular IL‐2 expression. *n* = 11 volunteers assessed for ST, PA and PB reactivity, *n* = 8 volunteers assessed for iNTSTy reactivity. One measurement was performed per volunteer. Mean ± SEM and individual responses are shown. Data were analysed by a paired Student's *t*‐test; *P*‐values are displayed.

The T_CM_ population (CD62L^+^, CD45RA^−^) is classically associated with homing to central lymphoid organs, with high proliferative potential and functional IL‐2 expression, supporting growth, expansion and survival of antigen‐specific memory T cells. We observed that CD8^+^ T_CM_ and CD4^+^ T_CM_ cells elicited after *S*. Typhi challenge are induced against *S*. Typhi and cross‐reactive only with PA (Figure 5a and b). Notably, there were no significant induced T_CM_ responses cross‐reactive against PB and iNTSTy.

**Figure 6:**
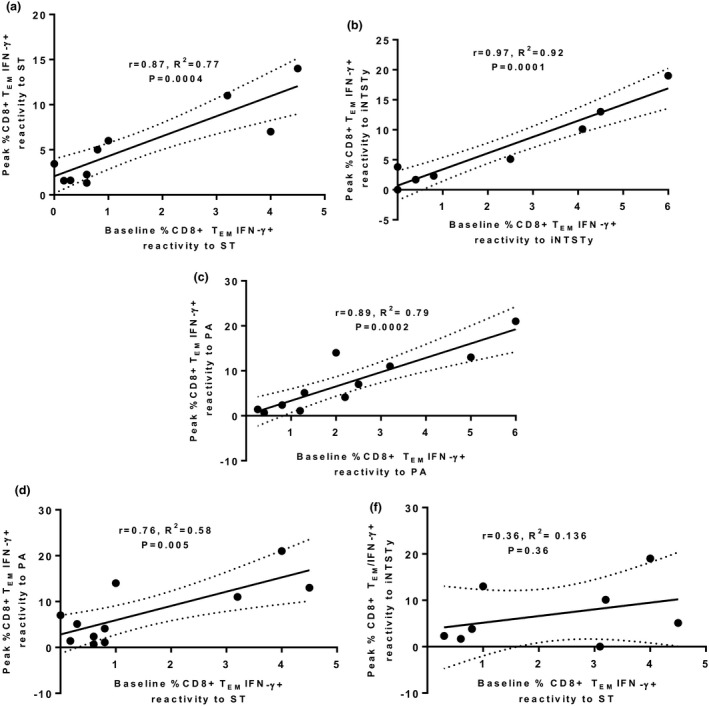
The magnitude of *Salmonella* Typhi baseline CD8^+^ T_EM_ cell IFN‐γ responses correlates with the magnitude of peak CD8^+^ T_EM_ cell IFN‐γ responses against *S*. Typhi and *Salmonella* Paratyphi A but not iNTSTy. PBMCs obtained prior to and after oral *S*. Typhi challenge at days 14, 21, 28 or 60 were stimulated with targets infected with either ST, PA, PB or iNTSTy. Individual peak CD8^+^ T_EM_ cell IFN‐γ responses postchallenge were determined for each serovar and are plotted against the corresponding baseline CD8^+^ T_EM_ cell IFN‐γ reactivity. Shown are baseline to peak CD8^+^ T_EM_ cell IFN‐γ reactivity for ST **(a)**, iNTSTy **(b)** and *S*. Paratyphi A **(c)**. Individual volunteer paired reactivity measurements are plotted, *n* = 11 for **a** and **c**, and *n* = 8 for **b**. **(d)** Correlation of baseline ST CD8^+^ T_EM_ cell IFN‐γ responses to peak CD8^+^ T_EM_ cell IFN‐γ responses to PA (paired responses, *n* = 11) **(e)** Correlation of baseline ST CD8^+^ T_EM_ cell IFN‐γ to peak CD8^+^ T_EM_ cell IFN‐γ responses to iNTSTy (paired responses, *n* = 8). *r*, *R* ^2^, and *P*‐values are presented.

Given the magnitude of the induced CD8^+^ T_EM_ subset after human *S*. Typhi challenge, and the identification of cross‐reactive multifunctional IFN‐γ^+^ CD8^+^T_EM_, we examined the relationship of pre‐existing frequencies of IFN‐γ^+^ CD8^+^ T_EM_ reactive against different invasive *Salmonella* strains to peak IFN‐γ^+^ CD8^+^ T_EM_ responses after human *S*. Typhi challenge. We observed that ST, PA and iNTSTy all had strong correlations in magnitude of frequencies of IFN‐γ^+^ CD8^+^ T_EM_ cells at baseline with the magnitude of the peak of the IFN‐γ^+^ CD8^+^ T_EM_ cell response against the respective invasive strain after oral *S*. Typhi challenge (Figure 6a–c), suggesting that *S*. Typhi challenge expands baseline reactive CD8^+^ T cells as part of the response to infection. Baseline IFN‐γ^+^ CD8^+^ T_EM_ cell reactivity to ST also correlated with the peak magnitude of the IFN‐γ^+^ CD8^+^ T_EM_ cell response against PA (Figure 6d). Notably, however, there was no correlation between baseline IFN‐γ^+^ CD8^+^ T_EM_ cell responses to ST and the peak magnitude of the IFN‐γ^+^ CD8^+^ T_EM_ cell iNTSTy response (Figure 6e) after *S*. Typhi challenge, indicating that volunteers with low or high IFN‐γ^+^ CD8^+^ T_EM_ cell baseline frequencies against ST did not have a predictable IFN‐γ^+^ CD8^+^ T_EM_ cell peak response against iNTSTy. This pattern suggests that a proportion of the cross‐reactive responses that are elicited after human oral *S*. Typhi challenge and that are reactive against iNTSTy are not expanded from the pre‐existing repertoire of CD8^+^ T_EM_ cells reactive to ST, underscoring differences in the properties of the cross‐reactive T‐cell responses to iNTSTy and PA.

We evaluated whether expression of the gut mucosal‐associated homing integrin α4β7 differed among the multifunctional *Salmonella* cross‐reactive peripheral CD8^+^ T_EM_ subsets observed after oral *S*. Typhi challenge, as evaluated at the peak of the blood CD8^+^T_EM_ cell IFN‐γ response against ST. We observed no differences in the frequencies of integrin α4β7 expression among CD8^+^ T_EM_ cells expressing two or more, or three or more concomitant functions, among the different invasive *Salmonella* (Supplementary figure 2). Additionally, there were no differences in cross‐reactive frequencies of CD8^+^ T_EM_ cells containing simultaneous effector functions of IFN‐γ^+^/TNF‐α^+^/CD107a^+^/MIP‐1β^+/−^ that also expressed integrin α4β7 (Supplementary figure 2). Hence, while the expression of α_4_β_7_ was noted in only a subset of the reactive multifunctional CD8^+^ T_EM_ cells, there was no difference in frequencies among multifunctional subsets induced after human oral *S*. Typhi challenge, and no significant differences in frequencies of α_4_β_7_ multifunctional CD8^+^ T_EM_ cell cross‐reactive with PA, PB or iNTSTy.

**Figure 7:**
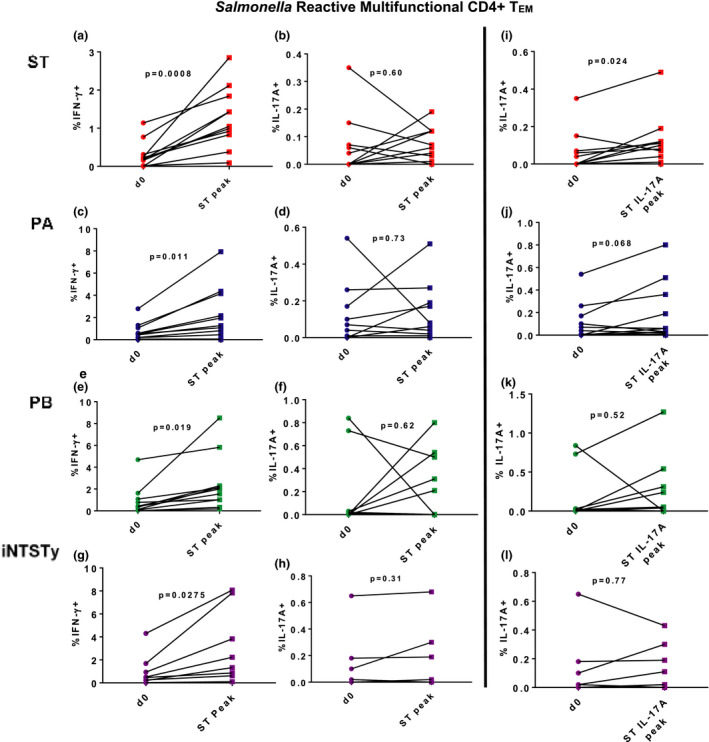
Differential kinetics, magnitude and frequencies of multifunctional, serovar cross‐reactive CD4^+^ Th1 and CD4^+^ Th17 effector memory cells in peripheral blood after human oral *wt* *Salmonella* Typhi challenge. PBMCs collected from volunteers prior to *S*. Typhi challenge (d0) or at the timepoint of peak blood total CD4^+^ T_EM_ IFN‐γ responses after *S*. Typhi challenge (ST peak) or peak peripheral CD4^+^ T_EM_ IL‐17A responses (ST peak IL‐17A) were stimulated with targets infected with either ST **(a, b, i)**, PA **(c, d, j)**, PB **(e, f, k)** or iNTSTy **(g–i)**. CD69^+^ CD4^+^ T_EM_ cells were analysed for simultaneous expression of six effector functions (production of TNF‐α, IL‐17A, IFN‐γ, MIP‐1β, IL‐2 and/or expression of CD107a). Multifunctional Th1 or Th17 cells were defined as expressing IFN‐γ (Th1) or IL‐17A (Th17) and at least one other additional effector function. Multifunctional CD69^+^/IFN‐γ^+^ CD4^+^ T_EM_ **(a, c, e, g)** or CD69^+^/IL‐17A^+^ CD4^+^ T_EM_ **(b, d, f, h)** cells were evaluated at the peak of the total CD4^+^ T_EM_ cell IFN‐γ response. Multifunctional CD69^+^/IL‐17A CD4^+^ T_EM_ cells were also evaluated at the peak of the total CD4^+^ T_EM_ cell IL‐17A response **(i–l)**. *n* = 11 volunteers assessed for ST, PA and PB reactivity, *n* = 8 volunteers assessed for iNTSTy reactivity. Paired responses are indicated by lines. One measurement per volunteer was made per timepoint. Data were analysed by a paired Student's *t*‐test; *P*‐values are displayed.

We also assessed whether serovar cross‐reactive multifunctional CD4^+^ T cells secreting IFN‐γ (Th1) or IL‐17A (Th17) cells are elicited after human oral *S*. Typhi challenge. High frequencies of multifunctional CD4^+^ T_EM_ cells, concomitantly expressing at least IFN‐γ and one or more additional effector functions, were elicited after oral *wt* *S*. Typhi challenge, as assessed at the peak of the total IFN‐γ CD4^+^ T_EM_ cell response against ST, and these responses were similarly cross‐reactive against PA, PB and iNTSTy (Figure 7a, c, e and g). Notably, at the peak of the CD4^+^ T_EM_ cell IFN‐γ response there were no significantly induced multifunctional Th17 responses against any of the invasive *Salmonella* strains (Figure 7b, d, f and h). More individuals had a peak peripheral blood CD4^+^ T_EM_ cell IL‐17A response at earlier timepoints after oral *S*. Typhi challenge than those observed for the peak peripheral blood CD4^+^ T_EM_ cell IFN‐γ response (Figure 1b and e). When we measured multifunctional Th17 induction at the peak of the Th17 response against ST (ST IL‐17A peak), we observed that significant frequencies of multifunctional Th17 with reactivity against ST were induced (Figure 7i). Interestingly, these lower magnitude peripheral multifunctional Th17 responses elicited after oral *wt* *S*. Typhi challenge were not significantly cross‐reactive against PA, PB or iNTSTy (Figure 7j–l).

**Figure 8:**
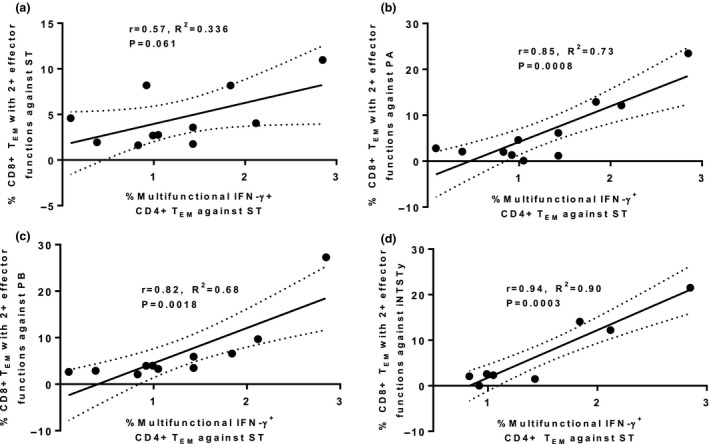
Induction of multifunctional Th1 against *Salmonella* Typhi after human oral challenge correlates with the induction of multifunctional serovar cross‐reactive CD8^+^ T_EM_ cells. PBMCs from individual volunteers at the peak of the total peripheral CD4^+^ T_EM_ IFN‐γ response were analysed for the frequencies of IFN‐γ^+^CD4^+^ T_EM_ cells with at least one or more concomitant additional effector functions (either CD107a expression or TNF‐α, MIP‐1β, IL‐17A or IL‐2 production) against ST and plotted against the frequencies of CD8^+^ T_EM_ cells, as evaluated at the peak of total peripheral CD8^+^ T_EM_ cell IFN‐γ response per volunteer, with at least 2 or more effector functions (production of TNF‐α, MIP‐1β, IL‐2, IL‐17A and/or expression of CD107a) against either ST **(a)**, PA **(b)**, PB **(c)** or iNTSTy **(d)**. Eleven volunteers were studied in **a–c** and 8 volunteers in **d**. Single paired measurements were made for each volunteer and plotted. Responses of individual volunteers are shown with correlation coefficient (*r*), coefficient of determination (*R* ^2^) and *P*‐value.

Multiple studies have demonstrated that CD4^+^ T cells are required for the generation of optimal CD8^+^ T‐cell memory cell responses and that repeated antigenic stimulation does not substitute for CD4^+^ T‐cell help when priming CD8^+^ T‐cell memory responses.^32^ Given the sizable frequencies of multifunctional serovar cross‐reactive Th1 elicited after human oral *S*. Typhi challenge in the peripheral blood, we examined the hypothesis that robust induced multifunctional CD4^+^ Th1 responses against ST correlates with peak multifunctional CD8^+^T_EM_ responses against ST and other invasive *Salmonella*. We observed that while there was a moderate positive relationship between the peak IFN‐γ^+^ CD4^+^ T_EM_ cell response against ST and the multifunctional CD8^+^ T_EM_ cell response (*P* = 0.061), this relation was not statistically significant (Figure 8a). However, we observed that the induced CD4^+^ T_EM_ cell response against ST correlated strongly with the cross‐reactive multifunctional CD8^+^ T_EM_ cell response against PA, PB and iNTSTy (Figure 8b–d). These data suggest a relationship between the induction of robust Th1 responses after *S*. Typhi challenge and the induction of high frequencies of cross‐reactive multifunctional CD8^+^ T_EM_ cells against invasive *Salmonella* strains in humans.

## Discussion

There are no vaccines in clinical use against *S*. Paratyphi or iNTS infection, despite significant disease burden and mortality. Phylogenetic studies show *S*. Typhi more closely related to PA than to iNTS *S*. Typhimurium strains.^7^ Biologic differences between iNTS *S*. Typhimurium and NTS *S*. Typhimurium impact antigen‐presenting cell migration in murine infection and human macrophage inflammatory and antibacterial cytotoxic responses.^33^, ^34^ Virulence factors, such as the Vi capsule, are present in *S*. Typhi but absent in PA and iNTS strains, and attachment pili differ considerably among serovars.^35^ In a murine model of invasive *Salmonella* vaccination, effective host memory defence responses were serovar‐dependent and LPS‐independent after oral challenge with different nontyphoidal Salmonella serovars.^36^ Recent studies have shown that very minor serovar differences in *Salmonella* immunodominant antigens PhoN and CdtB impact CD4^+^ T‐cell cross‐reactivity from T‐cell clones expanded after human *wt* *S*. Typhi challenge.^37^ Given genetic and phenotypic differences between *Salmonella* strains, the present study evaluates the development and functional specificity of adaptive heterologous T‐cell memory responses in humans against genetically distinct *Salmonella* sharing capacity for mucosal invasion and dissemination. Our methods emphasise assessment of T‐cell functionality upon exposure to antigen‐presenting cells infected with ST, PA, PB or iNTSTy, without selective expansion of memory cell clones, and on the characterisation of both human CD4^+^ and CD8^+^ T‐cell memory compartments induced after experimental oral *wt* *S*. Typhi challenge.

Among individuals orally challenged with *wt* *S*. Typhi and developing typhoid disease, we observed that the peripheral blood T‐cell response continues to evolve in most volunteers long after antibiotic treatment, with generation of peak peripheral blood CD4^+^ and CD8^+^ T_EM_ cells with different effector functions by 1 month or later postchallenge in most volunteers. The observation of peak peripheral human T memory cell responses occurring several weeks after clinical symptoms of typhoid disease contrasts with studies of different human viral mucosal infections, where elicitation of peripheral blood T cells with an effector memory phenotype peaks 1 week after development of symptoms in hantavirus infection^38^ or 1 week after intranasal challenge with influenza.^39^ In contrast to a variety of human viral infections, where systemic T‐cell responses appear much earlier in the clinical course of infection, the evolution of peripheral blood T‐cell effector memory responses against *S*. Typhi has a delayed kinetics.^40^, ^41^ One explanation for the observed delayed T_EM_ presence in the blood after *S*. Typhi challenge may be the result of T_EM_ cells migrating to mucosal sites initially during early infection and therefore present at reduced levels in circulation soon after infection. Alternately, the delayed peripheral blood T memory cell response may be consistent with early immune evasion mechanisms operative in invasive *S*. Typhi infection, whereby particular *S*. Typhi gene products impair antigen presentation to T cells^42^, ^43^ or impair recognition or inflammatory pathways by antigen‐presenting cells or mucosal tissues.^44^, ^45^ Attenuated *S*. Typhi vaccine Ty21a is currently administered as multiple doses of vaccine over a 1‐week period. Our observation of a delayed T‐cell effector response after *S*. Typhi challenge may support the benefit of a late timepoint booster immunisation for next‐generation live‐attenuated *S*. Typhi vaccination, after the peripheral memory T‐cell pool has matured in response to infection in humans, to potentially extend the duration of long‐lived immunity.

Multifunctional T‐cell responses are associated with superior immune protection in human infection with viruses^46^, ^47^, ^48^, ^49^ and intracellular bacteria and parasites.^50^, ^51^, ^52^ We have previously shown that individuals who resist development of typhoid disease after *S*. Typhi challenge have significantly increased frequencies of *S*. Typhi‐reactive multifunctional CD8^+^ T cells in peripheral blood prior to challenge relative to those who develop typhoid disease.^24^ These baseline T‐cell memory frequencies observed in individuals with no prior history of prior *S*. Typhi exposure or vaccination may be the result of exposure to other cross‐reactive Enterobacteriaceae or cross‐reactive environmental antigens.^53^ Here, we observed that high frequencies of multifunctional CD8^+^ T effector memory cells with two or more concomitant effector functions are induced after *S*. Typhi challenge and are highly cross‐reactive with PA, PB, as well as iNTSTy‐infected targets. Significant induction of highly multifunctional CD8^+^ T effector memory cells with three or more effector functions, however, was only observed against PB‐infected targets. This observation suggests that the heterologous CD8^+^ T‐cell responses induced in humans after *S*. Typhi challenge may involve a signal threshold for multifunctional responses that are not achieved from exposure to PA or iNTSTy‐infected targets. One hypothesis for why this may occur is that antigens presented by PA or iNTSTy‐infected targets may not interact as strongly with CD8^+^ T‐cell receptors, subsequently impacting downstream signalling and cytokine production, resulting in cross‐reactive T cells with fewer measured effector functions. Alternately, the immunodominant antigens or their peptide sequences may significantly differ between PA, PB and iNTSTy, leading to different frequencies of multifunctional CD8^+^ T‐cell responses. These studies are the first to demonstrate that multifunctional CD8^+^ T_EM_ cells produced after human *wt S*. Typhi exposure produces significantly induced cross‐reactive responses against PA, PB and iNTSTy, yet the functional characteristics of the CD8^+^ T_EM_ cell response are impacted, at least in part, by serovar.

Human CD8^+^ T‐cell cytolytic activity against *S*. Typhi‐infected targets, induced after Ty21a vaccination, is MHC I‐dependent^25^ and is very highly correlated with frequencies of IFN‐γ producing *S*. Typhi‐specific CD8^+^ T cells.^26^ In a murine model of vaccination against invasive *Salmonella*, CD8^+^ T cells and IFN‐γ signalling were important in recall host defence mechanisms on challenge with *S*. Typhimurium, impacting survival.^54^ Here, we demonstrate that the IFN‐γ producing CD8^+^ T_EM_ cells induced after human *S*. Typhi infection is primarily multifunctional, as we observed no significant induction of CD8^+^ T_EM_ IFN‐γ monofunctional cells. Additionally, there was no observed induced response by cross‐reactive CD8^+^ T effector memory single positive IFN‐γ‐producing cells against other invasive *Salmonella* strains. Hence, human challenge with *S*. Typhi leads to the induction of multifunctional serovar cross‐reactive IFN‐γ^+^ CD8^+^ T effector memory cells, given our observation of significantly induced CD8^+^ T_EM_ cells with the IFN‐γ^+^/TNF‐α^+^/CD107a^+^/MIP‐1β^+/−^ phenotype. Since IFN‐γ monofunctional T cells contrast significantly at the level of transcription from IFN‐γ multifunctional cells as observed in different human infections,^55^ our data suggest after human infection with *S*. Typhi, there is preferential induction of multifunctional CD8^+^ T_EM_ cells within the memory compartment, and only multifunctional IFN‐γ^+^CD8^+^ T_EM_ cell responses are significantly cross‐reactive.

A specific population of CD8^+^ T_EM_ cells with simultaneous evidence of degranulation (as measured by CD107a expression) and production of IFN‐γ and TNF‐α, with or without production of MIP‐1β, was highly induced after human *S*. Typhi challenge. This multifunctional population with 3 or more specific effectors was highly cross‐reactive with PA and PB but not iNTSTy. In contrast, human Ty21a vaccination led to the development of multifunctional CD8^+^ T_EM_ cells expressing IFN‐γ and CD107a without TNF‐α expression that were cross‐reactive against PB but not against PA.^31^ These data suggest that genetic changes in Ty21a, which lead to phenotypic changes including its attenuation and absence of Vi capsule for example, impact aspects of human antigen presentation and CD8^+^ T‐cell memory programming and that *wt* *S*. Typhi infection promotes a more robust cross‐reactive CD8^+^ T‐cell response against PA. In contrast to cross‐reactivity observed against PA after human *S*. Typhi challenge, this cross‐reactivity is markedly decreased when assessed against targets infected with the more genetically distant iNTSTy. Genetic and phenotypic differences between Ty21a and *wt* *S*. Typhi may account for the more limited CD8^+^ T‐cell cross‐reactive responses against PA observed after Ty21a vaccination. Our findings underscore the potential for reengineering *S*. Typhi‐based oral vaccines to obtain optimal cross‐reactive CD8^+^ T‐cell responses against PA in vaccination.

We observed strong correlations between frequencies of *S*. Typhi‐specific IFN‐γ^+^ CD8^+^ T_EM_ cells prior to challenge and the magnitude of the peak *S*. Typhi‐specific IFN‐γ^+^ CD8^+^ T_EM_ cell response after challenge. This observation suggests that part of the immune response to infection involves expansion of baseline T‐cell memory responses from the pre‐existing repertoire in individuals with no history of prior *S*. Typhi infection or vaccination. We also observed a correlation in PA and iNTSTy baseline IFN‐γ^+^ CD8^+^ T_EM_ cell responses to peak responses, suggesting that the pre‐existing IFN‐γ^+^ CD8^+^ T_EM_ cell repertoire reactive to these genetically distant invasive *Salmonella* are expanded by *S*. Typhi challenge. Interestingly, the relationship between baseline *S*. Typhi‐specific IFN‐γ^+^ CD8^+^ T_EM_ cell responses and peak PA IFN‐γ^+^ CD8^+^ T_EM_ cell responses also highly correlates, while the relationship to peak iNTS IFN‐γ^+^ CD8^+^ T_EM_ cell responses does not. These data suggest that baseline CD8^+^ T‐cell reactivity with iNTS corresponds predominantly to different CD8^+^ T‐cell antigen specificities, which are expanded after *S*. Typhi challenge, and that dominant CD8^+^ T‐cell antigens are different between iNTSTy and *S*. Typhi. These findings support observations by Napolitani *et al*., where more cross‐reactive T‐cell clones and the antigens they react with were shared between *S*. Typhi and PA rather than iNTS *S*. Typhimurium.^37^ Alternately, IFN‐γ^+^ CD8^+^ T cells induced by *S*. Typhi challenge may have differential activation to iNTS antigens compared to typhoidal serovars, secondary to potential differences in antigen presentation.

CD8^+^ T_EM_ cells induced after *S*. Typhi challenge expressing two or more effector functions, three or more effector functions, or the CD107a^+^/TNF‐α^+^/IFN‐γ^+^/MIP‐1β^+/−^ phenotype, did not differ in expression of gut‐homing molecule integrin α4β7 upon exposure to antigen‐presenting cells infected with either *S*. Typhi, PA, PB or iNTSTy. These data suggest that multifunctional, serovar cross‐reactive CD8^+^ T_EM_ cells generated after human *S*. Typhi challenge have similar potential for homing to gut mucosal tissues. That a high proportion of these induced multifunctional CD8^+^ T cells do not express integrin α4β7 supports similar data we have previously reported in volunteers immunised with attenuated *S*. Typhi vaccines.^31^, ^56^, ^57^ These results underscore the increased presence in circulation of memory T cells elicited in *S*. Typhi‐challenged adults with the ability to home to mucosal and other systemic tissues, which may prevent dissemination during future *Salmonella* infections.

We observed significant differences among memory T‐cell compartments with regard to T‐cell serovar cross‐reactivity. *Salmonella* Typhi challenge induced CD4^+^ and CD8^+^ T_CM_ producing IL‐2, but only, surprisingly, demonstrating significant cross‐reactivity against PA and not against iNTSTy‐ or PB‐infected targets. In some models of infection with intracellular bacteria or parasites, the T_CM_ population plays an essential role in host defence.^58^, ^59^ Thus, the absence of cross‐reactivity against iNTSTy or PB may impact vaccine design. In addition, after *wt* *S*. Typhi challenge, multifunctional CD8^+^ T_EMRA_ cells, terminally differentiated effector memory cells implicated in protection mechanisms in viral infections such as HIV and influenza,^60^, ^61^ were not significantly cross‐reactive against PA, PB or iNTSTy. The clinical significance of the absence of heterologous T‐cell responses within these memory compartments is unclear, but demonstrates that T‐cell memory subset reactivation is specifically and differentially impacted by serovar, potentially through differences in bacterial phenotype during infection of antigen‐presenting cells, or through specific differences in antigens between serovars, leading to differential T‐cell subset cross‐reactivity.

We show that the predominant multifunctional CD4^+^ T_EM_ cell response in human blood after *S*. Typhi challenge is a Th1 response, rather than a Th17 response. The induced multifunctional Th1 response is notably cross‐reactive against PA, PB and iNTSTy. In contrast, while a multifunctional induced Th17 response occurs against *S*. Typhi after challenge, this response is generally of lower magnitude than the Th1 response and is notably not cross‐reactive against iNTSTy, PA or PB. The role of Th17 immunity specifically in human host defence against *S*. Typhi or other typical invasive *Salmonella* strains is unclear. Studies with a nontyphoidal *S*. Typhimurium strain in a SIV‐infected macaque model of infection showed importance of Th17 responses in limiting bacterial dissemination from the gut mucosa.^62^ In a cohort of Bangladeshi patients with natural *S*. Typhi infection, T cells reactive with *S*. Typhi antigens were found to express IFN‐γ early after developing *S*. Typhi bacteraemia, while T cells producing IL‐17 reactive with *S*. Typhi antigens were only observed in significant numbers 2–4 weeks after bacteraemia development.^63^ Human immunisation with Ty21a generates IL‐17A‐producing CD4^+^ T cells at the terminal ileum mucosa, as well as CD8^+^ T cells producing IL‐17A at the mucosa and in the blood.^64^, ^65^, ^66^ The dominance of multifunctional CD4^+^ Th1 effector memory elicited in the blood in humans after oral *S*. Typhi challenge is consistent with findings demonstrating a prominent IFN transcriptional response in humans infected with *S*. Typhi^67^, ^68^ and may impact on the diversity of IFN‐γ‐dependent cytolytic mechanisms against *Salmonella*.^69^

We demonstrate that the magnitude of multifunctional IFN‐γ CD4^+^ T_EM_ cell response to *S*. Typhi correlates with induction of cross‐reactive multifunctional CD8^+^ T_EM_ cells in humans. Expansion and maintenance of memory CD8^+^ T cells are CD4^+^ T‐cell‐dependent in numerous infection models,^32^, ^70^, ^71^, ^72^, ^73^ with data supporting an essential role of CD4^+^ T cells in aspects of initial memory CD8^+^ T‐cell priming through factors such as CD27 and CD40 signalling.^74^, ^75^ We have previously shown that humans challenged with *S*. Typhi exhibited enhanced CD8^+^ T_EM_ cell responses *in vitro* when T regulatory cells are depleted during the process of stimulation with *S*. Typhi‐infected antigen‐presenting cells.^76^ Given that a major risk factor in humans for invasive *Salmonella* infection, particularly with the nontyphoidal serovars, is HIV infection and CD4^+^ T‐cell deficiency, these data underscore one mechanism whereby CD4^+^ T‐cell responses could impact a critical effector mechanism against invasive *Salmonella* infection in humans.

These data further the hypothesis that CD4^+^ T cells enhance immunity to invasive *Salmonella* in humans, at least in part, through priming memory CD8^+^ T‐cell responses and/or enhancing their activation upon secondary exposure to invasive *Salmonella* antigens. We show here that heightened multifunctional CD4^+^ T‐cell IFN‐γ responses after *S*. Typhi challenge correlate specifically with heterologous, serovar‐independent CD8^+^ T‐cell multifunctional responses upon secondary stimulation with invasive *Salmonella* antigens, pointing to the capability of *S*. Typhi in generating heterologous multifunctional CD4^+^ and CD8^+^ T‐cell responses in humans. Collectively, these data suggest that *S*. Typhi, as a parent strain for a vaccine for iNTS or *S*. Paratyphi strains, induces cross‐reactive multifunctional effector memory CD4^+^ T and CD8^+^ T‐cell responses. However, significant gaps in cross‐reactive immune responses are noted in T‐cell memory compartments and by specific cellular functionalities, dependent on invasive *Salmonella* strain, demonstrating that ultimately genetic differences between strains impact memory T‐cell recognition, activation and signalling against invasive *Salmonella*‐infected cells. Particularly for iNTS, these data underscore the importance of strain‐specific vaccination to prevent invasive *Salmonella* infection in humans.

## Methods

### Participants and human challenge model

Data were collected from clinical samples obtained from a human challenge model of typhoid fever (UKCRN ID 9297; approval: Oxfordshire Research Ethics Committee A [10/H604/53], as previously described by Waddington *et al*.^77^ Male or female volunteers, healthy, between ages 18 and 60, were recruited by the Oxford Vaccine Group, and written informed consent was obtained for participation. Individuals were excluded from the study if they had a history of prior *S*. Typhi vaccination and prior typhoid disease, or had resided in an *S*. Typhi endemic area for more than 6 months. Volunteers underwent challenge with a single oral dose of ~ 1.98 × 10^4^ CFU [range (1.5 × 10^4^–2.69 × 10^4^)] wild‐type *S*. Typhi (Quailes strain), suspended in sodium bicarbonate solution, administered after 90 min of fasting. The challenge study was conducted in an ambulatory setting with daily blood culture and daily surveillance for safety, clinical measurements and laboratory measurements. Participants received a two‐week course of antibiotics (oral ciprofloxacin) starting immediately at time of diagnosis of typhoid disease (TD) or by 14 days after challenge. TD was defined as either (1) one or more positive blood cultures collected after day 5 postchallenge or (2) sustained fever observed after challenge (temperature above 38°C lasting 12 or more hours). PBMCs from 11 participants who developed TD were randomly selected for further characterisation of memory T‐cell immune responses. A summary of demographic and clinical parameters of participants whose samples were used in this study is provided in Supplementary table 1; these parameters are similar to those observed in the broader study cohort as previously described.^77^

### Preparation of effector cells

Peripheral blood mononuclear cells were separated by gradient centrifugation and cryopreserved in liquid nitrogen within 4 h of blood draw. On thawing, cell viability was assessed by trypan blue exclusion. Cells were rested overnight at 37°C, 5% CO_2_ in RPMI containing 100 U mL^−1^ penicillin, 100 µg mL^−1^ streptomycin, 50 µg mL^−1^ gentamicin, 1 mm l‐glutamine, 10 mm HEPES buffer and 10% heat‐inactivated foetal bovine serum, prior to presentation to infected stimulator cells.

### Preparation of stimulator cells and Salmonella infection of stimulator cells

Autologous Epstein–Barr virus (EBV)‐transformed B‐lymphoblastoid cell lines (B‐LCL) were generated from PBMCs from individual volunteers, to ultimately serve as stimulator cells, as previously described.^78^ *Salmonella* strains were obtained from University of Maryland‐CVD reference stocks: *S*. Typhi strain ISP‐1820 (a clinical isolate from Chile, Vi+), *S*. Paratyphi A strain CV 223 (ATCC #9150, Manassas, VA, USA) and *S*. Paratyphi B strain CV 23 (a clinical isolate from Chile), and the *S*. Typhimurium invasive nontyphoidal strain D65, with the ST313 genotype (a clinical isolate from a bacteraemic child in Mali^79^). B‐LCL cells were infected with individual *Salmonella* strains (at a 10:1 bacteria:B‐LCL cell ratio) in RPMI without antibiotics. Infection proceeded for 3 h, after which time cells were washed in cRPMI, and incubated overnight in the presence of 150 µg mL^−1^ gentamicin. To confirm B‐LCL infection with *Salmonella* strains, infected B‐LCL were stained with FITC‐conjugated antibody against *Salmonella* common structural Ag (CSA‐1, Kierkegaard & Perry, Gaithersburg, MD, USA) and analysed by flow cytometry.

### *Ex vivo* stimulation of effector cells

B‐LCL‐infected stimulator cells were gamma‐irradiated (6000 rad) and co‐cultured with PBMCs. PBMCs cultured without target cells and with uninfected target cells were used as negative controls, and PBMCs cultured in the presence of Staphylococcal enterotoxin B (SEB) at 10 µg mL^−1^ served as a positive control. *Ex vivo* stimulation proceeded in the presence of FITC‐conjugated anti‐CD107a (BD Biosciences, San Jose, CA, USA). After 2 h, monensin (1 µg mL^−1^) and Brefeldin A (2 µg mL^−1^) were added to cocultures. Cells were incubated overnight and harvested for immunostaining.

### Surface and Intracellular staining, and gating protocol

Peripheral blood mononuclear cells were stained for live/dead discrimination using yellow fluorescent viability dye (YEVID, Invitrogen, Carlsbad, CA, USA) and then washed with buffer (PBS with 2% FCS), and nonspecific Fc receptor binding was blocked by incubation with human immunoglobulin (3 µg mL^−1^; Sigma‐Aldrich, St. Louis, MO, USA) for 20 min at room temperature. Cells were then stained extracellularly and intracellularly with a panel of fluorochrome‐conjugated monoclonal antibodies as previously described.^24^ Cells were fixed in 1% paraformaldehyde and stored at 4°C until analysis. Flow cytometry was performed using an LSRII flow cytometer (BD), and during sample acquisition, between 300 000 and 500 000 events were collected per sample. Data were analysed using WinList version 9.0 (Verity Software House, Topsham, ME, USA). Singlet CD3^+^ CD4^+^ T cells or CD3^+^ CD8^+^ T cells and T memory subsets were evaluated for expression of CD45RA and CD62L for determination of T central memory (T_CM_; CD62L^+^ CD45RA^−^), T effector memory (T_EM;_ CD62L^−^CD45RA^−^) and T effector memory CD45 RA^+^ (T_EMRA_; CD62L^−^ CD45 RA^+^) subsets. Naïve T cells (T_N_) were defined as CD62L^+^ CD45RA^+^. The FCOM analysis tool was used to characterise events based on selected gate combinations in multidimensional space to determine multifunctional subsets. Flow cytometry experiments were performed at the Flow Cytometry and Mass Cytometry Core Facility of the University of Maryland School of Medicine Center for Innovative Biomedical Resources (CIBR).

### Statistics

All statistical tests were performed with GraphPad Prism 6.0 software (GraphPad Prism, La Jolla, CA, USA). Comparisons between two groups were performed with a paired Student's *t*‐test. Pearson's correlations were calculated assuming bivariate Gaussian distributions among variables, and linear regression lines were fit to the data with 95% confidence bands. Statistical significance was accepted for *P* < 0.05.

### Study approval

In obtaining the clinical samples used in this study, written informed consent was obtained, and the clinical protocol was approved by National Research Ethics Service, Oxfordshire Research Ethics Committee A (10/H0604/53). The clinical study proceeded in accordance with the International Conference on Harmonisation Good Clinical Practice Guidelines.

## Author contributions

RRR, RW and MBS: Conceptualization; design of the experiments. RRR and SF: Conduction of the experiments. RRR, RW and MBS: Data analysis. SF, JB, TCD, CJ, CSW, MML, AJP and MBS: Contribution to reagents/materials/analysis/tools. RRR, RW, TCD, AJP, MML and MBS: Writing of the paper. TCD, CJ, CSW, MML and AJP: Set up of challenge model; clinical data generation; collection and processing of the PBMCs specimens used in this study.

## Conflicts of Interest

AJP is chair of the UK Department of Health's Joint Committee on Vaccination and Immunisation and the European Medicine Agency Scientific Advisory Group on Vaccines, and a member of WHO's Strategic Advisory Group of Experts and an NIHR Senior Investigator. The other authors declare no conflicts of interest.
