# Supplementary material for: Human Salmonella Typhi exposure generates differential multifunctional cross‐reactive T‐cell memory responses against Salmonella Paratyphi and invasive nontyphoidal Salmonella
Source: Clin Transl Immunology. 2020 Sep 24;9(9):e1178. doi: 10.1002/cti2.1178 (PMC7512505; doi:10.1002/cti2.1178)
Supplement: Supplementary file 1 [file CTI2-9-e1178-s001.pdf]

SUPPLEMENTARY MATERIALS

**Mean Age (years)**	29.18 (range, 19-44)
**Gender Female/Male (%)**	36/64
**Ethnicity (%) African/Afro-Caribbean Caucasian**	9<br>91
**Time to TD (hours)**	191.5 (range, 134-248)
**Maximum temperature (Celsius)**	38.6 (range 37.5-40.0)
**Maximum CRP (mg/L)**	65.4 (range, 12.9-138.5)
**Drop in Hgb (g/dL)**	2.65 (range, 1.1-4.4)
**Drop in Platelets ($X10^3$ /µL)**	71.4 (range, 0-122)
**Drop in WBC ($X\ 10^3$ cells/µL)**	3.18 (range, 1.27-6.9)

Supplementary Table 1: Demographic and clinical parameters of oral wt *S*. Typhi challenged volunteers. Volunteers (n=11) randomly selected from a larger cohort (as previously described by Waddington *et.al.* (reference 77)) challenged orally with wt *S*. Typhi develop typhoid disease (TD) on average 8 days after mucosal infection. Volunteers develop fever and other systemic signs characteristic of typhoid disease, including anemia,leukopenia, and thrombocytopenia near the time of TD, as previously described.[77] CRP (C-reactive protein), Hgb (Hemoglobin), WBC (White Blood Cell count). Average values and range are presented for continuous variables above.

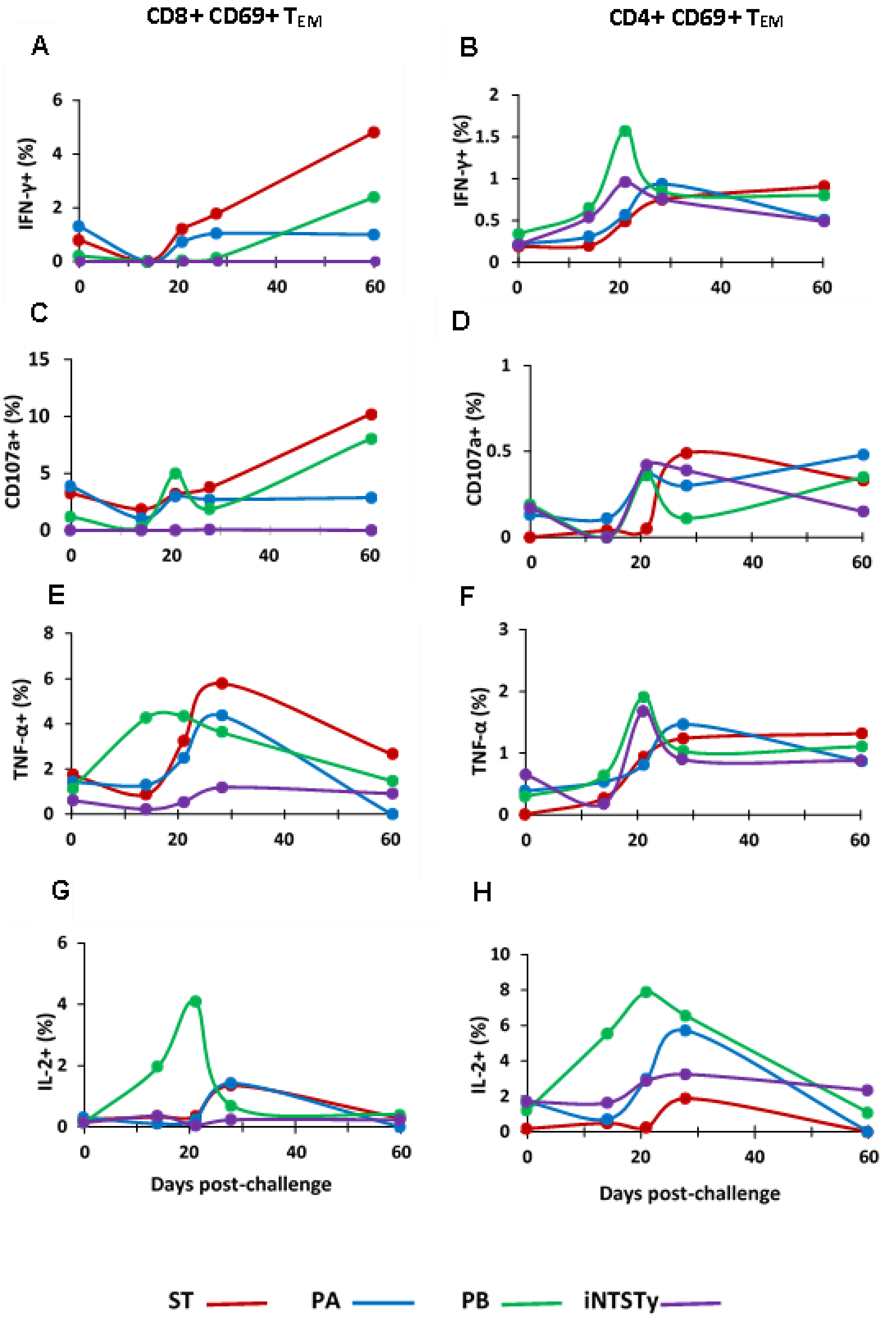
CD8+ CD69+ T_{EM}
CD4+ CD69+ T_{EM}
A
B
C
D
E
F
G
H
IFN-γ+ (%)
CD107a+ (%)
TNF-α+ (%)
TNF-α (%)
IL-2+ (%)
Days post-challenge
ST
PA
PB
iNTSTy

Supplementary Figure 1 (continued)

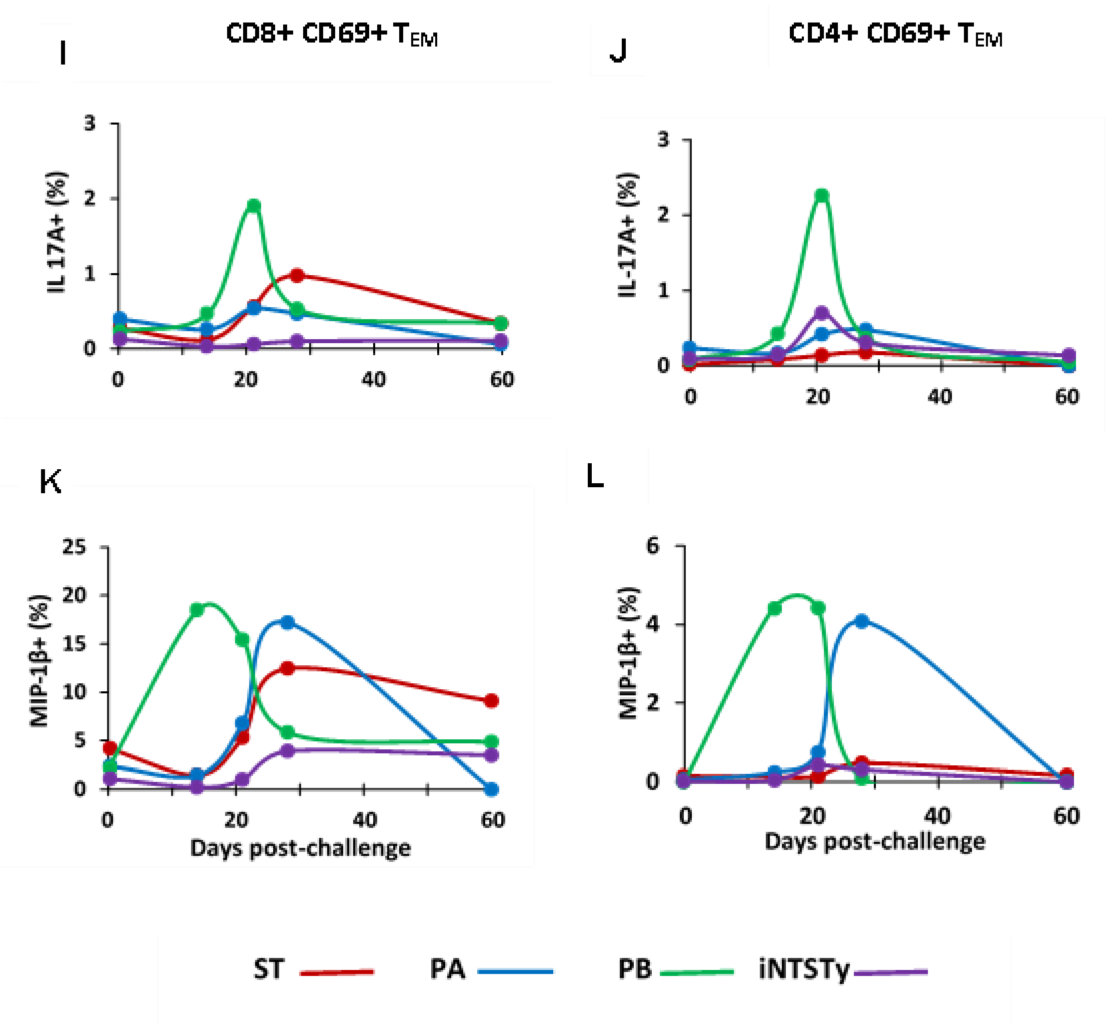

Supplementary Figure 1: Kinetics of *Salmonella* serovar cross-reactive T cell effector memory responses after oral *S*. Typhi challenge in a representative volunteer. PBMCs were collected prior to challenge, or on days 14, 21, 28, or 60 post-challenge, and were stimulated with targets infected with either *S*. Typhi (ST, red), *S*. Paratyphi A (PA, blue), *S*. Paratyphi B (PB, green), or invasive non-typhoidal *Salmonella* Typhimurium (iNTSTy, purple), and analyzed by flow cytometry. Background responses from uninfected targets were subtracted from target cell responses, and net responses are displayed. Each dot represents one measurement per timepoint, with timepoints interconnected by lines. Frequencies of CD8+ CD69+ T_{EM} (**A, C, E, G, I, K**) or CD4+ CD69+ T_{EM} cells (**B, D, F, H, J, L**) expressing either IFN-γ (**A, B**), CD107a (**C, D**), TNF-α (**E, F**), IL-2 (**G, H**), IL-17A (**I, J**), and MIP-1β (**K, L**), are shown over time.

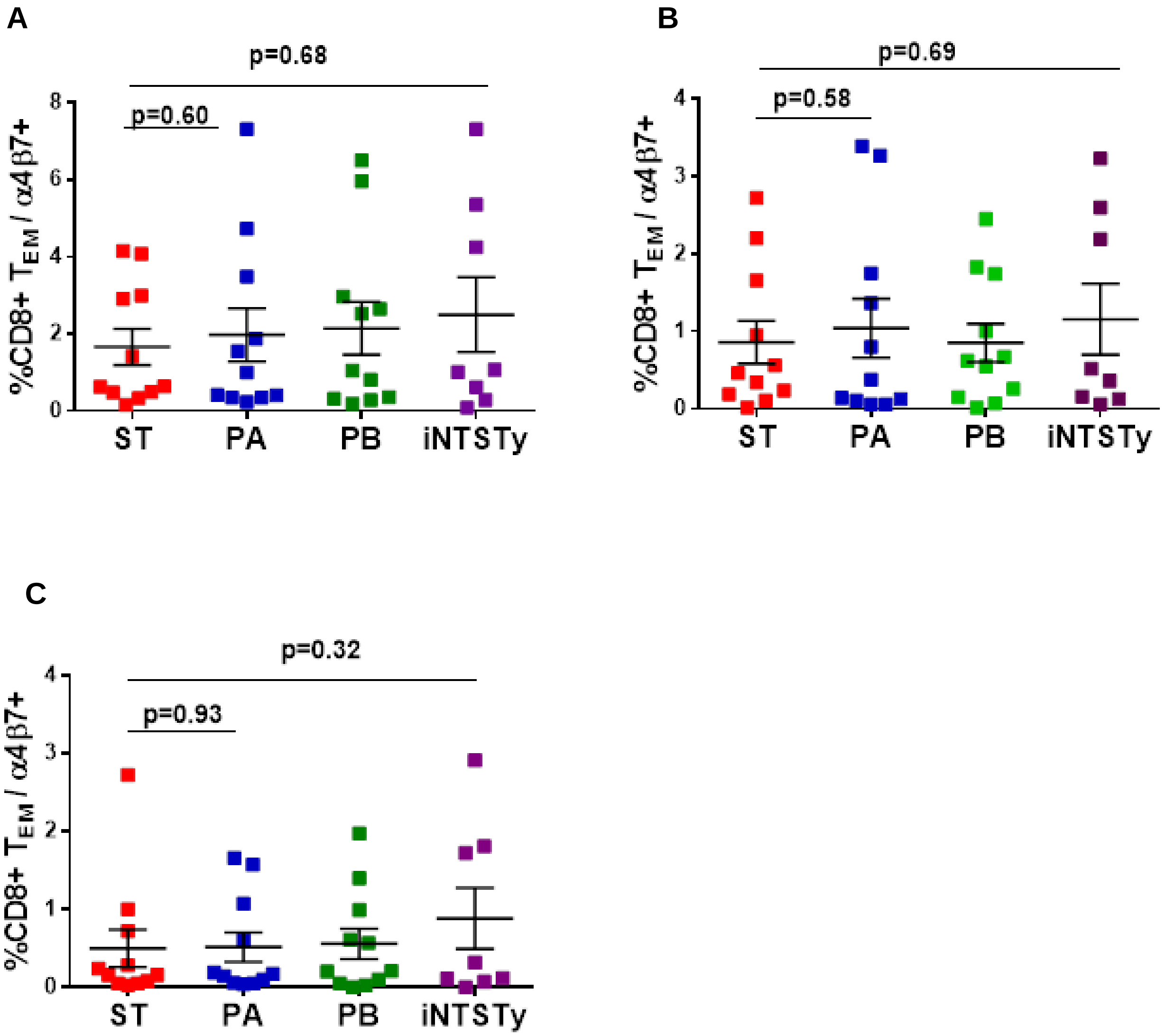

Supplementary Figure 2: Multifunctional cross-reactive blood CD8+ T_{EM} responses after *S.* Typhi challenge have similar frequencies of integrin α4β7 expression. PBMCs from volunteers challenged orally with *S*. Typhi were analyzed at the peak blood CD8+ T_{EM} IFN-γ response in each individual for expression of integrin α4β7 and effector functions of simultaneous production of IFN-γ, TNF-α, MIP-1β, IL-2, or surface expression of CD107a, after re-stimulation with either ST, PA, PB, or iNTS infected target cells. Mean +/- SEM and individual responses are shown. **A)** Frequencies of integrin α4β7+ CD8+ T_{EM} with 2 or more concomitant effector functions, **B)** Frequencies of integrin α4β7+ CD8+ T_{EM} with 3 or more concomitant effector functions, **C)** Frequencies of integrin α4β7+ CD8+ T_{EM} cells with simultaneous TNF-α, IFN-γ production and surface CD107a expression, with or without MIP-1β production. n=11 volunteers for ST, PA, PB reactivity, n=8 volunteers for iNTSTy reactivity. One measurement was performed per volunteer. Statistics were calculated by Paired Student's *t* test, *P* values are displayed.
